# Ribosome Profiling Reveals Genome-Wide Cellular Translational Regulation in *Lacticaseibacillus rhamnosus* ATCC 53103 under Acid Stress

**DOI:** 10.3390/foods11101411

**Published:** 2022-05-13

**Authors:** Xuejing Fan, Kenan Zhang, Zongcai Zhang, Zhen Zhang, Xue Lin, Xin Liu, Zhen Feng, Huaxi Yi

**Affiliations:** 1 Key Laboratory of Dairy Science, Ministry of Education, College of Food Science, Northeast Agricultural University, 600 Changjiang Road, Harbin 150030, China; 13019006983@163.com (X.F.); kn980909@163.com (K.Z.); zhangzongcai1999@163.com (Z.Z.); zhangzhen2245@163.com (Z.Z.); linlinlin_n@163.com (X.L.); liuxin1070306@163.com (X.L.); 2 Spice and Beverage Research Institute, Chinese Academy of Tropical Agricultural Sciences, Wanning 571533, China; 3 College of Food Science and Engineering, Ocean University of China, Qingdao 266100, China

**Keywords:** *L. rhamnosus*, translation regulation, transcription regulation, acid shock response, translation efficiency

## Abstract

During fermentation and food processing, *Lacticaseibacillus rhamnosus* ATCC 53103 can encounter many adverse conditions, and acid stress is one of them. The purpose of the present study was to investigate the influence of acid stress on the global translational and transcriptional regulation of *Lacticaseibacillus rhamnosus* ATCC 53103. Two pH values (pH 6.0 vs. pH 5.0) were applied, the effects of which were studied via ribosome profiling and RNA sequencing assay. Under acid stress, many genes showed differential changes at the translational and transcriptional levels. A total of 10 genes showed different expression trends at the two levels. The expression of 337 genes—which mainly participated in the ABC transporters, amino acid metabolism, and ribosome functional group assembly pathways—was shown to be regulated only at the translational level. The translational efficiency of a few genes participating in the pyrimidine and amino acid metabolism pathways were upregulated. Ribosome occupancy data suggested that ribosomes accumulated remarkably in the elongation region of open reading frame regions under acid stress. This study provides new insights into *Lacticaseibacillus rhamnosus* ATCC 53103 gene expression under acid stress, and demonstrates that the bacterium can respond to acid stress with synergistic translational and transcriptional regulation mechanisms, improving the vitality of cells.

## 1. Introduction

In recent years, *Lacticaseibacillus* (formerly *Lactobacillus*) *rhamnosus* Gorbach Goldin (*L. rhamnosus* GG) has been widely used in fermented food and dairy products, such as yogurt, fermented coffee, juice, etc. [1,2,3,4,5,6]. During fermentation and food processing, the continuous accumulation of organic acid causes a decrease in environmental pH, which affects the synthesis of nucleic acids and proteins, the degradation of substrates, and the utilization of nutrients, and also inhibits the growth and metabolism of cells [7,8]. Many acid adaptation mechanisms have been developed in lactic acid bacteria (LAB), such as the proton-translocating ATPase, regulatory factors, and transcription factors, which have been used to maintain cytoplasmic pH homeostasis and improve the resistance ability of LAB under acid stress [9,10,11]. Previous studies on the effect of acid stress on LAB have solely investigated the regulation of gene expression at the transcriptional level [12,13]. At present, translational regulations have been widely demonstrated to be involved in cells’ responses to environmental stresses, and mRNA translation plays an important role in gene expression regulation, as has also has increasingly recognized by researchers [14]. Meanwhile, the way in which LAB regulates gene expression at the translational level in response to acid stress is not fully understood.

Ribosomal profiling (Ribo-Seq) addresses the need for global expression assessment by estimating the ribosome occupancy and single-codon resolution, and quantifying the translation to the genome-wide level. This can precisely delineate translated regions and reveal the full coding potential of the genome [15,16]. Ribosome-protected mRNA fragments (RPFs) and total mRNAs can be quantified simultaneously by sequencing. The distribution of RPF reads illustrates ribosome occupation, while their abundance reveals the ribosome density. They are also used to measure the translational efficiency (TE), which ultimately determines the protein expression level [17,18]. Even since the advent of ribosome profiling, this important and powerful technique has been widely used in many studies of cellular activities in various organisms under different environmental stresses, such as the decrease in the number of RPFs (21 nt) and the translational regulation of *Saccharomyces cerevisiae* in response to high osmotic pressure and oxidative stresses [19], or the adaptation of yeasts’ responses to oxidative and amino acid starvation stresses [14,20]. The ribosomal footprints gathered in the open reading frames (ORFs) could increase the viability of *Escherichia coli* under heat stress [21]. Studies have also been conducted on the translational regulation of retinal pigment epithelium cells’ responses to hypoxic stress [22], and the translational regulation of *Eubacterium limosum* under autotrophic growth conditions [23]. However, the study of translational regulation in LAB has scarcely been reported.

In this study, simultaneous Ribo-Seq and RNA-Seq were used to establish quantitative translational and transcriptional regulation of *L. rhamnosus* ATCC 53103 under acid stress. The results showed that about 40% of identified differentially expressed genes were shared by the translational and transcriptional levels, and the ribosomal occupancy of the AS group was higher than that of the CG group in the elongation region of ORFs, which affected the TE of *L. rhamnosus* ATCC 53103 under acid stress. These results provide preliminary insights and new strategies for increasing the viability of *L. rhamnosus* under acid stress.

## 2. Materials and Methods

### 2.1. Strain, Culture Conditions, and Acid Stress

*L. rhamnosus* ATCC 53103 was obtained from the American Type Culture Collection (Manassas, VA, USA). The strain was revitalized in de Man, Rogosa, and Sharpe (MRS, HuanKai Microbial Sci. and Tech. Co., Ltd., Guangdong, China) medium at 37 °C. For acid stress, the cells were collected via centrifugation when the OD_600_ reached 1.8 (6000× *g*, 10 min, 4 °C). Then, the strain with the inoculation of 2% was resuspended in MRS medium (pH 5.0). The OD_600_ and the cell number of the cultures were 0.212 and 6.09 log_10_CFU/mL at t_0_, respectively. The growth of the strain was measured with an automatic growth curve analyzer (Bioscreen C Pro; OY Growth Curves, Helsinki, Finland) at 600 nm (OD_600_).

### 2.2. Total RNA Extraction and Library Construction

Cultures were harvested by centrifugation when the OD_600_ reached 1.0 (6000× *g* for 15 min at 4 °C). Total RNA was extracted, and the library was constructed as described in a previous study [24].

### 2.3. Ribo-Seq

The RPFs were prepared and the Ribo-Seq library was constructed as described in a previous study [24]. We added chloramphenicol (200 µg/mL) to the cultures and shook them for 2 min when the OD_600_ reached 1.0. The cells were washed using 20 mL of resuspension buffer and collected via centrifugation. Mixing the cells with cell lysis buffer and incubating them on ice for 10 min, the cells were triturated and the lysate was collected. Then, 5 µL of DNase I (Roche) and 7.5 µL of RNase I (Abgene) were added to 300 µL of lysate to cultivate for 45 min, and 10 µL of RNase inhibitor (Ambion) was added to the lysate to stop the digestion. RPFs were isolated using the Concentrator-25 Kit (R1017, Zymo Research, Orange County, CA, USA) and RNAClean. The Ribo-Seq library was constructed using the NEBNext^®^ Multiple Small RNA Library Prep Set for Illumina^®^ (catalog no. E7300S, E7300L). The 140–160 bp PCR products were enriched to generate a cDNA library, and sequenced using Illumina HiSeq^TM^ 2500.

### 2.4. Analysis of Differentially Expressed Genes

The gene expression level was normalized using the fragments per kilobase of transcript per million (FPKM) mapped reads method to eliminate the influence of different gene lengths and the amount of sequencing data on the calculation of gene expression. The edgeR package (http://www.rproject.org/ 28 December 2020) was used to ascertain differentially expressed genes (DEGs). Gene Ontology (GO) annotation and the Kyoto Encyclopedia for Genes and Genomes (KEGG) pathways of DEGs were analyzed. DEGs were divided into five groups as described in a previous study [24]: unchanged, homodirectional, opposite, translational, and transcriptional.

### 2.5. Analysis of DEGs’ Translational Efficiency, and Correlation with DEGs’ Expression

The analysis of the TE of all DEGs was carried out using RiboDiff [24,25]. According to the TE of the DEGs, and their expression at the transcriptional level, all DEGs were divided into five groups: homodirectional, opposite, TE, transcriptional, and unchanged.

### 2.6. Data Analysis

Raw data were filtered as described in a previous study [26]. The trimmed reads were mapped to the reference transcriptomic data of *L. rhamnosus* (https://www.ncbi.nlm.nih.gov/nuccore/NC_013198.1 28 December 2020). Retained reads were aligned, and DEGs were identified and calculated as described in a previous study [24]; we chose genes with a false discovery rate (FDR) < 0.05 and ∣log_2_(fold change)∣ > 1, which were defined as DEGs.

## 3. Results

### 3.1. Effects of Acid Stress on DEGs’ Expression and Function

**Figure 1:**
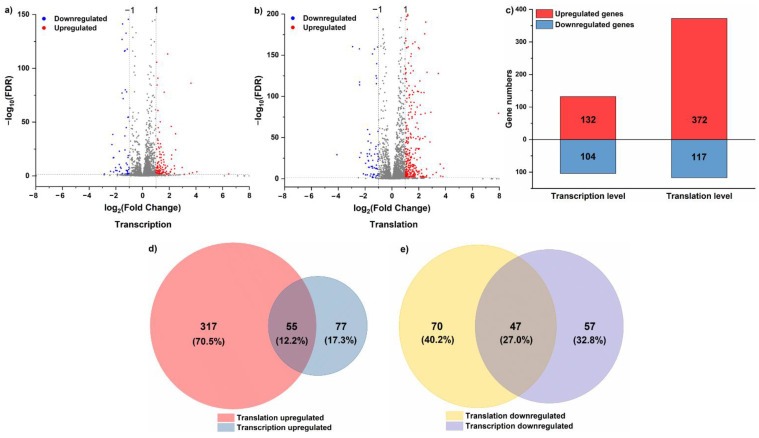
(**a**) The volcano map of DEGs in *L. rhamnosus* ATCC 53103 at the transcriptional level under acid stress (∣log_2_fold change∣ > 1 and FDR < 0.05). (**b**) The volcano map of DEGs in *L. rhamnosus* ATCC 53103 at the translational level under acid stress (∣log_2_fold change∣ > 1 and FDR < 0.05). (**c**) The number of DEGs in *L. rhamnosus* ATCC 53103 under acid stress at the transcriptional or translational levels. (**d**) The relationships between upregulated DEGs at these two levels. (**e**) The relationships between downregulated DEGs at these two levels.

**Figure 2:**
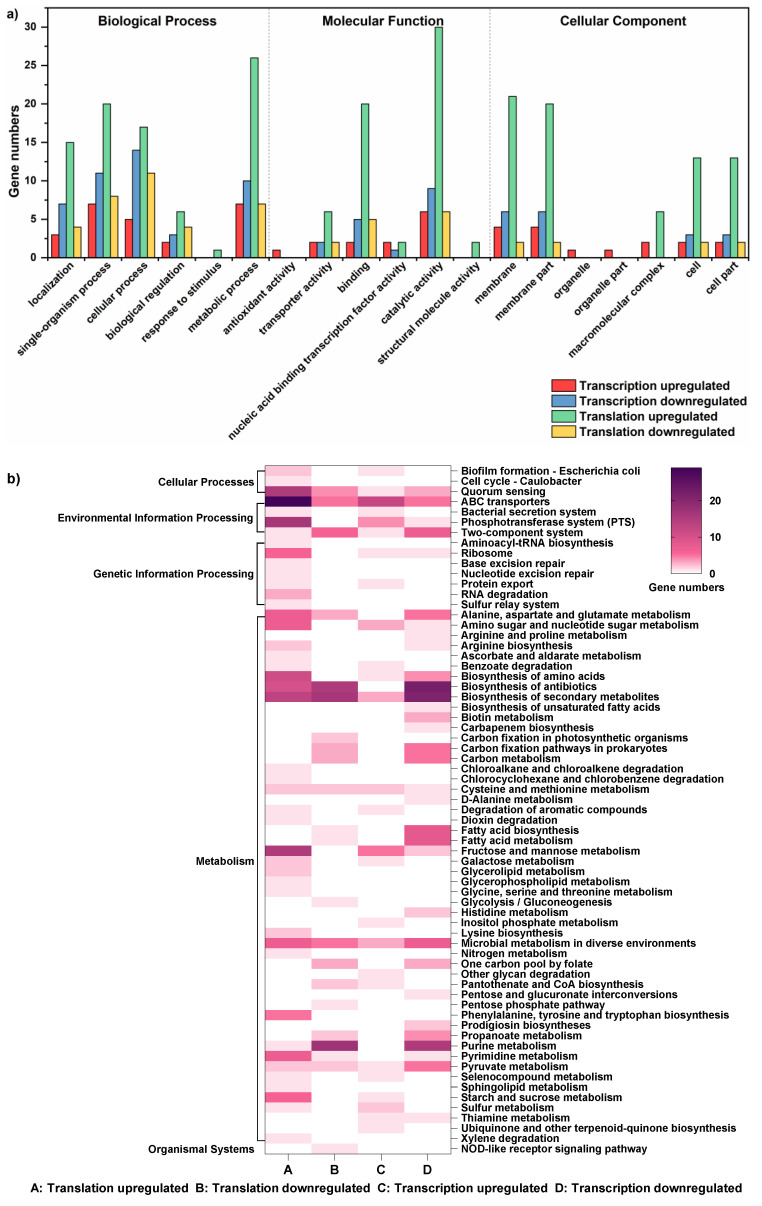
(**a**) GO analysis of DEGs in the AS and CG groups at the translational and transcriptional levels. (**b**) Significantly enriched KEGG pathways of DEGs in the AS and CG groups at the translational and transcriptional levels.

The regulation of translation and transcription under acid stress was monitored by sequencing of RPFs and total mRNAs in the control group (CG, pH 6.0) and the acid stress group (AS, pH 5.0). Gene expression changes induced by acid stress at the translational and transcriptional levels are shown in Figure 1. There were 132 and 372 upregulated DEGs at the transcriptional and translational levels, respectively, and there were 104 and 117 downregulated DEGs at these two levels, respectively (Figure 1a–c). Around 40% of the regulated genes were shared between the translational and transcriptional levels, including 12.2% of upregulated genes and 27.0% of downregulated genes (Figure 1d,e). GO annotation and KEGG pathway analysis of DEGs at these two levels were performed (Figure 2). The results indicated that DEGs largely overlapped in GO at the two levels. Most DEGs were prominently upregulated in GO at the translational level, and were mainly involved in metabolic and single-organism processes, catalytic activity, and the membrane components (Figure 2a). Many KEGG pathways appeared to largely overlap, such as ABC transporters, quorum sensing, two-component systems, biosynthesis of amino acids, and metabolism of purine pathways. However, large numbers of KEGG pathways did not overlap at these two levels, such as aminoacyl-tRNA biosynthesis and RNA degradation pathways (Figure 2b).

### 3.2. Effects of Acid Stress on the Dynamic Profiles of Translation and Transcription

**Figure 3:**
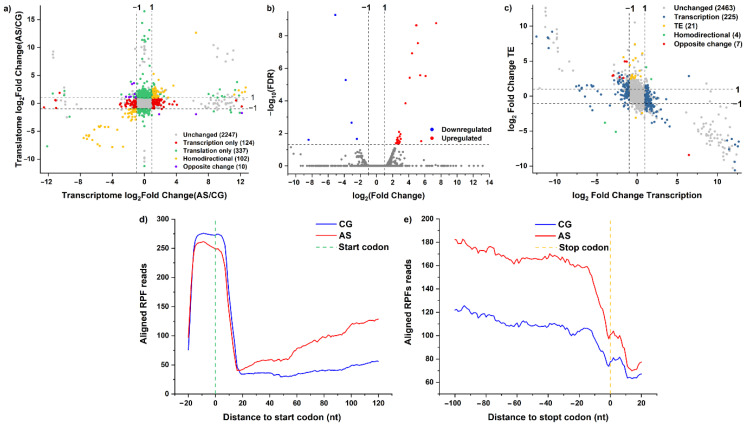
(**a**) The gene expression changes at the translational and transcriptional levels simultaneously in *L. rhamnosus* ATCC 53103 under acid stress. (**b**) The volcano plot of the TE responses of DEGs under acid stress. (**c**) Simultaneous monitoring of the distribution of log_2_fold change TE and corresponding log_2_fold change mRNA abundance in *L. rhamnosus* ATCC 53103 under acid stress. The four dashed lines indicate log_2_fold change values of −1 or +1. (**d**) The density of ribosomal footprints around the start codon under acid stress. (**e**) The density of ribosomal footprints around the stop codon under acid stress.

To access the overall changes in DEGs’ expression trends at the two levels simultaneously, DEGs were classified into distinct groups (Figure 3a).

There were 102 co-regulated DEGs at these two levels, and their expression had similar trends (yellow dots); 55 of these DEGs were co-upregulated, and 47 were co-downregulated. These DEGs mainly participated in the purine metabolism, ABC transporters, two-component system, pyrimidine metabolism, and biosynthesis of amino acids pathways. The DEGs *purQ*, *purS*, *purM*, *purN*, *purH*, and *purD* were downregulated by 193.03-, 151.33-, 57.73-, 47.84-, 16.14-, and 6.32-fold at the transcriptional level, respectively; they also were downregulated from 50- to 200-fold at the translational level. They mainly participated in the purine metabolism pathway. The DEGs *dltB*, *dltD*, *bceA*, and *agrA* were also downregulated by about 2- to 3-fold at the two levels, respectively; they mainly participated in the two-component system pathway.

There were 10 co-regulated DEGs at these two levels, and their expression had opposite trends (blue dots). *ArgG* was upregulated by 2.11-fold at the translational level and downregulated by 2.14-fold at the transcriptional level; it participated in the amino acid biosynthesis pathway. *CitC* was upregulated by 3.18-fold at the translational level and downregulated by 2.33-fold at the transcriptional level; it participated in the two-component system pathway. *PyrR1* was upregulated by 10.95-fold at the translational level and downregulated by 2.89-fold at the transcriptional level; it participated in the pyrimidine metabolism pathway.

There were 124 regulated DEGs at the transcriptional level only (red dots). These DEGs participated in the fatty acid metabolism, microbial metabolism in diverse environments, biosynthesis of secondary metabolites, and pyruvate metabolism pathways. The DEGs *accA*, *accC1*, *fabZ*, *accB*, *fabF*, *bkr2*, and *pksA* were each downregulated by about 2-fold. They mainly participated in the fatty acid metabolism pathway.

There were 337 regulated DEGs at the translation level only (green dots). The DEGs *dppE*, *livH*, *braF*, *tcyJ*, *ftsX*, *potD*, *nlpA*, *dppE*, *gbuA*, and *oppA* were upregulated, and they mainly participated in the ABC transporter pathway. The DEGs *trpF*, *trpC*, and *trpD* were upregulated by 14.51-, 12.16-, and 107.45- fold, respectively, and they mainly participated in the tyrosine, phenylalanine, and tryptophan biosynthesis pathways. The DEGs *carB*, *carA*, *pyrB*, *gltB*, *argH*, and *mtnU* were upregulated by 3.32-, 4.48, 11.59-, 4.11-, 4.53-, and 3.02-fold, respectively, and they participated in the alanine, aspartate, and glutamate metabolism pathways. The DEGs *rpsP*, *rpmI*, *rplQ*, *rplR*, *rpsH*, and *rpmH* were upregulated, and they mainly participated in the assembly of ribosome functional groups. Many DEGs participated in the phosphotransferase system (PTS), fructose, mannose, pyrimidine, starch, sucrose, amino sugar, nucleotide sugar metabolism, and microbial metabolism in diverse environments pathways; these DEGs also were upregulated. Meanwhile, the DEGs *coaD*, *ppdK*, and *ldh* participated in metabolic pathways, and they were each downregulated by about 2-fold. *mprF*, encoding lysyl-tRNA synthetase, was also downregulated by only 2.32-fold.

### 3.3. Effects of Acid Stress on DEGs’ Translational Efficiency

With FDR < 0.05 and∣log_2_FC∣ > 1, the TE of 27 DEGs was upregulated, while the TE of 5 DEGs was downregulated (Figure 3b and Table 1). The KEGG pathway enrichment analysis determined that these DEGs primarily participated in the amino acid, pyrimidine, and purine metabolism pathways, as well as the biosynthesis of secondary metabolites pathway. The DEGs *trpF* and *trpD* participated in the tyrosine, phenylalanine, and tryptophan biosynthesis pathways, while the DEGs *carA*, *pyrB*, and *argH* participated in the alanine, aspartate, and glutamate metabolism pathways. The DEGs *pyrC* and *pyrR1* participated in the pyrimidine metabolism pathway. The DEG *acpP* participated in the biosynthesis of secondary metabolites pathway. The TE of these DEGs was upregulated. Meanwhile, the DEGs *purD* and *purH* participated in the biosynthesis of purine metabolism pathway, and their TE was downregulated (Table 2).

### 3.4. Effects of Acid Stress on the Dynamic Profiles of Translational Efficiency and Transcription

Based on the expression of genes at the transcriptional level and the regulation of TE, the DEGs were divided into five groups (Figure 3c). There were 21 DEGs that were not regulated at the transcriptional level; however, their TE was changed significantly. The DEGs *carA*, *pyrB*, and *argH* participated in the alanine, aspartate, and glutamate metabolism pathways, and their TE was upregulated. The DEGs *trpF* and *trpD* participated in the tyrosine, phenylalanine, and tryptophan biosynthesis pathways, and their TE was also upregulated. The TE of *pyrC* and *accp* was upregulated under acid stress; these DEGs mainly participated in the metabolism of pyrimidine and the biosynthesis of secondary metabolites, respectively. The DEGs *hsp18* and *HSP17.6C* encode heat-shock proteins, and their TE was upregulated. The TE of *fmnP* was also upregulated; this gene encodes predicted membrane proteins.

There were 225 DEGs that were only regulated at the transcriptional level; however, their TE was not changed significantly. The DEGs *accA*, *accC1*, *accD*, *fabZ*, *accB*, *fabF*, *bkr2*, and *pksA* participated in the biosynthesis and metabolism of fatty acids pathway, and their expression was downregulated significantly. The DEGs *purA*, *guaC*, *purK*, *purB*, *purN*, *purM*, *purF*, *purL*, *purQ*, *purS*, *purC*, *purK*, and *purE* mainly participated in the purine metabolism pathway, and their expression was downregulated significantly. The DEGs *dltB*, *dltC*, *dltD*, *citC*, *bceA*, and *agrA* mainly participated in the two-component system pathway; their expression was also downregulated. The DEGs *tauA*, *potA*, *potB*, *pstS1*, *ssuB1*, *macB2*, *nodI*, *ugpB*, *ugpA*, and *ugpC* mainly participated in the ABC transporter pathway, and their expression was upregulated. In addition, 22 DEGs were identified in the biosynthesis of secondary metabolites pathway, and they were all regulated only at the transcriptional level.

Only 4 DEGs’ expression had regulation trends similar to their TE. These included the DEGs *purD* and *purH*, involved in the purine metabolism and biosynthesis of secondary metabolites pathways, which were downregulated at the transcriptional level, and their TE was also downregulated. A total of 7 DEGs’ expression had opposite regulation trends to their TE. The DEGs *glnP* and *nodI* participated in the ABC transporter pathway, and their expression was downregulated at the transcriptional level; however their TE was upregulated. The expression of *pyrR1* was also downregulated at the transcriptional level, while its TE was upregulated; this gene was involved in the pyrimidine metabolism pathway.

### 3.5. Effects of Acid Stress on Ribosome Accumulation in the ORF Regions

In order to quantify the translational regulation, the ribosomes’ average occupancy was computed around the start and stop codons. The distribution of averaged reads in the 5’ and 3’UTRs is shown in Figure 3d,e. Two groups of footprints mapped to the initiation region showed a clear and significant increase, with the 5’ end roughly at the nucleotide −15 position. The averaged read occupancy of the CG was higher than that of the AS group. The results showed that acid stress did not reduce the averaged read occupancy of the ribosomes, which increased from the region of nucleotide 20 to 120. The average ribosome occupancy of the AS group at nucleotide 120 after the start codon was about double the reads of the CG. In addition, the average ribosome occupancy of the AS group at the nucleotides from −100 to −20 in the stop codon regions was about 50% higher than that of the CG.

## 4. Discussion

Not only were the changes of gene expression explored on a genome-wide scale, the interactions between the translational and transcriptional regulation were also revealed in *L. rhamnosus* ATCC 53103 under acid stress using Ribo-Seq and RNA-Seq. There were 488 regulated DEGs at the translational level, while the number of regulated DEGs at the transcriptional level was only 236. These results indicate that translational regulation can be a more rapid and direct means of acid stress response compared to transcriptional regulation, because no new mRNA needs to be produced [27]. Hence, translational regulation plays an integral role in acid stress responses for *L. rhamnosus* ATCC 53103. There were 337 regulated DEGs at the translational level only, which mainly participated in the ABC transporter, amino acids biosynthesis and metabolism, and ribosome assembly pathways. The translational regulation of ABC transporter genes could regulate the synthesis of ABC transporters, which mediate the active transport of molecules across the cell membranes, and this process is helpful for *L. rhamnosus* ATCC 53103 to resist acid shock rapidly and maintain intercellular homeostasis [28,29]. Amino-acid-dependent acid-tolerance systems have been approved as a sophisticated defense mechanism against damage caused by acid in LAB [30]. The upregulated expression of DEGs participating in the biosynthesis and metabolism of amino acids at the translational level may help to increase the production of ATP and sequester protons, which are important for pH homeostasis [31]. Based on the high-level synthesis of proteins and the requirements of growing cells, the formation of ribosomes in the bacteria is an important and efficiently regulated process. The assembly of an intact ribosome requires many complex and regulated functions, such as the coordinated synthesis of ribosomal proteins [32]. In this study, some DEGs involved in the assembly of ribosome functional groups were upregulated only at the translational level under acid stress. This might help the bacteria to adjust the number of ribosomes in proportion to the metabolic state and the growth rate of cells in order to meet the demand for protein synthesis under acid stress [32]. Hence, translational regulation appeared to play an important role in *L. rhamnosus* ATCC 53103 under acid stress.

About 40% of DEGs were shared between the translational and transcriptional levels. There were 112 co-regulated DEGs at the two levels, and they mainly participated in the purine metabolism, ABC transporter, two-component system, pyrimidine metabolism, and amino acid biosynthesis pathways. The DEGs *purH*, *purQ*, *purM*, *purN*, *purD*, and *purS* were downregulated at both levels, and they were mainly involved in purine metabolism. As the signal mediator, the downregulation of *pur* genes could help bacteria to save energy and accelerate the growth of cells under acid stress, indicating that purine metabolism involves the bacterial stringent response, and could conceivably enhance resistance to acid stress in *L. rhamnosus* ATCC 53103 [33]. Normally, the two-component system can help bacteria to respond to environmental stresses, because it contains a target gene expression regulator and a membrane-associated histidine kinase [21]. The overlap between transcriptional and translational regulation could increase the flexibility of gene expression, which could improve bacterial adaptability to acid stress [34].

TE is an important index that can be used to identify previously unknown translational events [35]. Only 21 DEGs were not regulated at the transcriptional level, and their TE was changed significantly under acid stress. These DEGs participated in the amino acid metabolism pathway, and their TE increased significantly. Amino acids participate in the physiological processes of *L. rhamnosus* ATCC 53103, such as intracellular pH regulation, stress resistance, protein synthesis, and metabolic energy generation [31]. Although the genes’ expression was not changed significantly at the transcriptional level, the upregulation of TE still improved the biosynthesis of amino acids, and further increased the tolerance under acid stress. Many DEGs participated in the purine metabolism, fatty acid biosynthesis and metabolism, and two-component system pathways; their expression was downregulated at the transcriptional level. These results are different from previous results that showed that these genes’ expression was upregulated under different environmental stresses [30], and the TE of these DEGs was not changed significantly, indicating that translational regulation might control the production of effector proteins in response to certain stresses [21]. Therefore, we hypothesized that translational regulation might play different roles in cells under different stresses, and that the TE remaining relatively constant would be helpful for *L. rhamnosus* ATCC 53103 to respond to acid shock, while the downregulated expression of related DEGs might help to reduce cells’ energy consumption [36].

ORFs are important mediators of transcript-specific translational control—especially from the upstream ORFs to their downstream coding sequences [22]. In this study, the averaged read occupancy of the CG was higher than that of the AS group around the start codons. This might have been due to the reduction in ribosome abundance at the initiation region, which could partially rescue protein production under acid stress [37]. Meanwhile, the average ribosome occupancy of the AS group was higher than that of the CG in the elongation region. Typically, upstream ORFs would reduce the TE of the main downstream ORFs under normal conditions [38]. Another study also reported that when the translational inhibition by an upstream ORF was reduced, the ribosome occupancy and TE of numerous mRNAs were increased in *Arabidopsis thaliana* under hypoxic stress [39]. Stress-induced repression of ribosomes and the reduction in the initiation rates of genes have been thought to be adaptive, because they not only increase the pool of free ribosomes and tRNAs, but also minimize resource waste. These responses could have direct benefits in increasing the elongation rate of genes, rescuing protein production, and promoting cell growth under stress [37].

## 5. Conclusions

The present work revealed the landscape of translational and transcriptional regulation in *L. rhamnosus* ATCC 53103 under acid stress by using Ribo-Seq and RNA-Seq. At the translational and transcriptional levels, about 40% of DEGs were co-regulated, indicating that the overlap between the translational and transcriptional regulations improved the adaptability of *L. rhamnosus* ATCC 53103 under acid stress. Many DEGs were regulated only at the transcriptional level; however, their TE was not changed significantly. For some DEGs, *L. rhamnosus* ATCC 53103 appeared to possess specialized mechanisms to offset or enhance the effects of transcriptional regulation under acid stress. The ribosomal footprints of the AS group were higher than those of the CG in the elongation region of the ORFs, which also maintained the TE in *L. rhamnosus* ATCC 53103 under acid stress. These results indicate that ORFs play an important role in the regulation of gene expression in response to acid stress; other mechanisms of the effects of ORFs still require further study.

## Figures and Tables

**Table 1:** Genes with differential translational efficiency regulation under acid stress.

Category	Gene_ID	Gene_Name	TE_CG	TE_AS	log_2_FC	FDR
Upregulated	LGG_RS00110	*--*	6.75	43.02	2.67	0.0396827
	LGG_RS00155	*--*	0.67	4.93	2.88	0.0153195
	LGG_RS00490	*trpF*	0.28	13.15	5.55	0.0303543
	LGG_RS00500	*trpD*	0.61	41.48	6.09	2.95 × 10^−^^6^
	LGG_RS00715	*TTE1650*	0.01	0.07	2.81	0.0409188
	LGG_RS02170	*--*	0.01	1.7	7.41	1.666 × 10^−^^9^
	LGG_RS02790	*TTE1650*	0.01	0.07	2.81	0.0409188
	LGG_RS03170	*HSP17.6C*	0.01	0.35	5.13	2.827 × 10^−^^8^
	LGG_RS04550	*TTE1650*	0.01	0.07	2.81	0.0409188
	LGG_RS04690	*--*	0.1	0.79	2.98	0.0112087
	LGG_RS04695	*nodI*	0.09	0.67	2.9	0.0327759
	LGG_RS06620	*fmnP*	0.24	1.47	2.61	0.0184817
	LGG_RS06985	*carA*	4.43	25.52	2.53	0.0251229
	LGG_RS06990	*pyrC*	1.76	12.29	2.8	0.0081897
	LGG_RS06995	*pyrB*	0.48	10.29	4.42	1.185 × 10^−^^7^
	LGG_RS07000	*pyrP*	0.38	11.49	4.92	2.265 × 10^−9^
	LGG_RS07005	*pyrR1*	0.15	4.68	4.96	2.265 × 10^−^^9^
	LGG_RS08210	*TTE1650*	0.01	0.07	2.81	0.0409188
	LGG_RS08915	*--*	0.86	4.82	2.49	0.0409188
	LGG_RS09795	*clpC*	1.15	19.98	4.12	3.804 × 10^−^^6^
	LGG_RS10200	*acpP*	5.56	33.81	2.6	0.0251229
	LGG_RS10665	*--*	1.94	10.39	2.42	0.0396827
	LGG_RS13400	*Cndp1*	1.92	15.49	3.01	0.0219037
	LGG_RS13405	*oppA*	1.72	73.85	5.42	2.659E-06
	LGG_RS13420	*hsp18*	0.78	9.47	3.6	0.0001423
	LGG_RS13465	*glnP*	0.02	0.12	2.58	0.0327759
	LGG_RS13475	*argH*	0.24	1.6	2.74	0.0278452
Downregulated	LGG_RS05995	*--*	903	2.64	−8.42	0.0251229
	LGG_RS08685	*purD*	15.22	0.44	−5.11	5.176 × 10^−^^10^
	LGG_RS08690	*purH*	22.29	1.58	−3.82	5.257 × 10^−^^6^
	LGG_RS09365	*oppA*	3.01	0.56	−2.43	0.0219037
	LGG_RS13815	*--*	40.17	4.72	−3.09	0.002218

Note: FC, fold change; TE, translational efficiency.

**Table 2:** DEG enrichment results of TE with KEGG pathway analysis.

Category	Pathway ID	Pathway Name	*p*-Value	Genes	Genes
Upregulated	ko00250	Alanine, aspartate, and glutamate metabolism	0.002	LGG_RS06985, LGG_RS06995, LGG_RS13475	*carA*, *pyrB*, *argH*
	ko00240	Pyrimidine metabolism	0.002	LGG_RS06985, LGG_RS06990, LGG_RS06995, LGG_RS07005	*carA*, *pyrC*, *pyrB*, *pyrR1*
	ko00400	Phenylalanine, tyrosine, and tryptophan biosynthesis	0.008	LGG_RS00490, LGG_RS00500	*trpF*, *trpD*
	ko00999	Biosynthesis of secondary metabolites—unclassified	0.029	LGG_RS10200	*acpP*
	ko01110	Biosynthesis of secondary metabolites	0.057	LGG_RS00490, LGG_RS00500, LGG_RS13475	*trpF*, *trpD*, *argH*
	ko01100	Metabolic pathways	0.068	LGG_RS00490, LGG_RS00500, LGG_RS06985, LGG_RS06990, LGG_RS06995, LGG_RS07005, LGG_RS13475	*trpF*, *trpD*, *carA*, *pyrC*, *pyrB*, *pyrR1*, *argH*
	ko00220	Arginine biosynthesis	0.071	LGG_RS13475	*argH*
	ko01230	Biosynthesis of amino acids	0.108	LGG_RS00490, LGG_RS00500, LGG_RS13475	*trpF*, *trpD*, *argH*
Downregulated	ko02024	Quorum sensing	0.151	LGG_RS09365	*oppA*
	ko00230	Purine metabolism	0.187	LGG_RS08685 LGG_RS08690	*purD*, *purH*
	ko02010	ABC transporters	0.432	LGG_RS09365	*oppA*

## Data Availability

Data is contained within the article.
